# Effects of Mountain Uplift and Climatic Oscillations on Phylogeography and Species Divergence of *Chamaesium* (Apiaceae)

**DOI:** 10.3389/fpls.2021.673200

**Published:** 2021-05-24

**Authors:** Hong-Yi Zheng, Xian-Lin Guo, Megan Price, Xing-Jin He, Song-Dong Zhou

**Affiliations:** ^1^Key Laboratory of Bio-Resources and Eco-Environment of Ministry of Education, College of Life Sciences, Sichuan University, Chengdu, China; ^2^Sichuan Key Laboratory of Conservation Biology on Endangered Wildlife, College of Life Sciences, Sichuan University, Chengdu, China

**Keywords:** *Chamaesium*, phylogeography, phylogeny, Himalayan-Hengduan Mountains, species distribution modeling

## Abstract

Exploring the effects of orographic events and climatic shifts on the geographic distribution of organisms in the Himalayas-Hengduan Mountains (HHM) region and Qinghai-Tibetan Plateau (QTP) is crucial to understand the impact of environmental changes on organism evolution. To gain further insight into these processes, we reconstructed the evolutionary history of nine *Chamaesium* species distributed across the HHM and QTP regions. In total, 525 individuals from 56 populations of the nine species were analyzed based on three maternally inherited chloroplast fragments (*rpl16*, *trnT-trnL*, and *trnQ-rps16*) and one nuclear DNA region (internal transcribed spacer, ITS). Fifty-two chloroplast DNA (cpDNA) and 47 ITS haplotypes were identified in nine species. All of the cpDNA and ITS haplotypes were species-specific. Phylogenetic analysis suggested that all nine species form a monophyletic clade with high support. Dating analysis and ancestral area reconstruction revealed that the ancestral group of *Chamaesium* originated in the southern Himalayan region at the beginning of the Paleogene (60.85 Ma). The nine species of *Chamaesium* then separated well during the last 25 million years started in Miocene. Our maxent modeling indicated the broad-scale distributions of all nine species remained fairly stable from LIG to the present and predicted that it will remain stable into the future. The initial split of *Chamaesium* was triggered by climate changes following the collision of the Indian plate with the Eurasia plate during the Eocene. Subsequently, divergences within *Chamaesium* may have been induced by the intense uplift of the QTP, the onset of the monsoon system, and Central Asian aridification. Long evolutionary history, sexual reproduction, and habitat fragmentation could contribute to the high level of genetic diversity of *Chamaesium*. The higher genetic differentiation among *Chamaesium* populations may be related to the drastic changes of the external environment in this region and limited seed/pollen dispersal capacity.

## Introduction

The Himalayan-Hengduan Mountains (HHM) are key features of the biodiversity hotspots of South and East Asia (Myers et al., 2000; Zhang et al., 2002; Marchese, 2015), with alpine plant diversity being a significant contributor to the hotspot (Wu et al., 2011). The Himalayas define the southern margin of the Qinghai-Tibetan Plateau (QTP), whereas the HHM of Southwest China forms the plateau’s southeastern frontier (Zhang et al., 2002). Previous studies have suggested that the HHM region was formed by the collision of the Indian plate with Eurasia and the consequent rise of the Himalayas and QTP (Dupont-Nivet et al., 2010; Zhang et al., 2012; Chatterjee et al., 2013). Despite many studies focusing on a younger age for collision (20–30 Mya or younger) (Zheng et al., 2000; Aitchison et al., 2007; Wan et al., 2007), the collision is widely accepted to have started ca. 55–50 Mya during the Eocene (Zhang et al., 2012; Chatterjee et al., 2013). During the uplift in the Miocene, the Himalayan basins Thakkhola, Gyirong, and Zhada reached a mean elevation of 4,000 or 6,000 m (Garzione et al., 2001; Rowley et al., 2001; Saylor et al., 2009). The QTP experienced further growth during the Late Miocene and the Pliocene (Li and Fang, 1999; Zheng et al., 2000; Mulch and Chamberlain, 2006), particularly at its eastern edge within the HHM. The paleobotanical and paleoclimatic data suggests that Hengduan Mts. reached peak elevation shortly before the Late Pliocene, and the orogeny of Hengduan Mts. occurred as a final propagation of the uplift after 10 Ma (Sun et al., 2011).

The uplift of the QTP caused a series of topographical and climatic changes, which possibly served as a major force in species diversification (Harmon et al., 2008; Qiu et al., 2011; Xu et al., 2015; Mosbrugger et al., 2018). For example, the rising QTP and adjacent mountainous areas formed barriers to atmospheric circulation in Asia during the Oligocene (Ruddiman and Kutzbach, 1991; Kutzbach et al., 1993; An et al., 2001), and consequently promoted the formation and development of the East Asian monsoon system (Sun and Wang, 2005; Huntington et al., 2006; Guo et al., 2008). In contrast, the progressive uplift of the Himalayas and the Tianshan, and the retreat of the Tethys Sea, in conjunction with global cooling may have contributed to the aridification of Central Asia since the Miocene (Tapponnier et al., 2001; Mulch and Chamberlain, 2006; Wu et al., 2007; Qiang et al., 2011). The creation of these distinct climatic zones had a significant influence on the distribution and evolution of plant taxa (Sun and Wang, 2005; Lowry et al., 2008; Zhang and Sun, 2011; Keller and Seehausen, 2012). Additionally, the Hengduan Mts. region has a unique mountainous topography and was impressive during the orogeny. This area is distinguished by north-south oriented high peaks separated by valley floors (Zhang et al., 2004; Cao et al., 2009; Sun et al., 2011; Song et al., 2016). Dramatic elevation variations ranging from ca. 1,000 m in some valleys to 7,556 m at the summit of Minya Konka can form “sky islands” (Lopez-Pujol et al., 2011; He and Jiang, 2014; Sklenar et al., 2014). These distinct habitat zones and ecosystems, such as the sky islands, also resulted in speciation and radiation (Shepard and Burbrink, 2008; Wang Y.J. et al., 2009; Xu et al., 2010; Qiu et al., 2011). Consequently, the climatic and orogenic changes caused by uplift have played a critical role in the origin, speciation and evolution of several plant taxa throughout the HHM and adjacent areas.

With such an intricate geological and ecological diversity, the HHM region is attractive for studying the drivers of species diversification and evolution. Previous phylogenetic and biogeographical studies have focused on species-level diversification resulting from the uplift of the QTP (Liu et al., 2006; Wang L.Y. et al., 2009). Phylogeographical analyses within species have demonstrated that the divergence and demography of populations have also been intensively affected by the rise of the QTP and the Quaternary climatic oscillations in this area (Yang et al., 2008, 2012; Wang et al., 2010; Li et al., 2011), with deep intraspecific divergences formed over a range of timescales (Wang L.Y. et al., 2009; Li et al., 2011). Among these studies, however, very few have focused specifically on an endemic genus of the HHM region (Cun and Wang, 2010; Opgenoorth et al., 2010). Therefore we aimed to determine species’ origins, diversification and origin of existing distribution patterns from phylogenetic, biogeographical and phylogeographical data of *Chamaesium* H. Wolff.

*Chamaesium* is the only genus of the *Chamaesium* clade in Apiaceae and is endemic to the HHM region, where it grows in alpine meadows, shrublands and forests above 3,000 m (Guo et al., 2018). It is characterized by having fruits with developed primary and secondary ribs and 1- pinnate leaf blade, which easily distinguishes it from other genera of Apiaceae. In addition, *Chamaesium* has unique morphological characteristics as it has fruits with twice as many ribs and vascular bundles as Apiaceae’s other genera. According to records of *Chamaesium* in the Flora of China and Guo et al. (2018), nine morphologically distinct species are accepted and the recent phylogenetic study (Zhou et al., 2009; Guo et al., 2018) indicated that this genus forms a distinct monophyletic clade, the *Chamaesium* clade. *Chamaesium* is thus an ideal taxon to investigate the evolution and diversification of alpine plants in the HHM region.

In this study, we reconstructed the phylogenetic tree of *Chamaesium* based on materials from 56 populations of nine species and used biogeographical and phylogeographical approaches to identify the mechanism of origin, divergence and population evolutionary history in the genus *Chamaesium*. Our aims were to: (1) reconstruct the phylogenetic relationships within the genus *Chamaesium* based on materials from 56 populations; (2) explore the origin and species diversification of *Chamaesium* throughout the HHM region and its adjacent areas (QTP); (3) explore the geological and climatic changes that influenced distribution shifts and genetic diversity of *Chamaesium*.

## Materials and Methods

### Sampling

**FIGURE 1:**
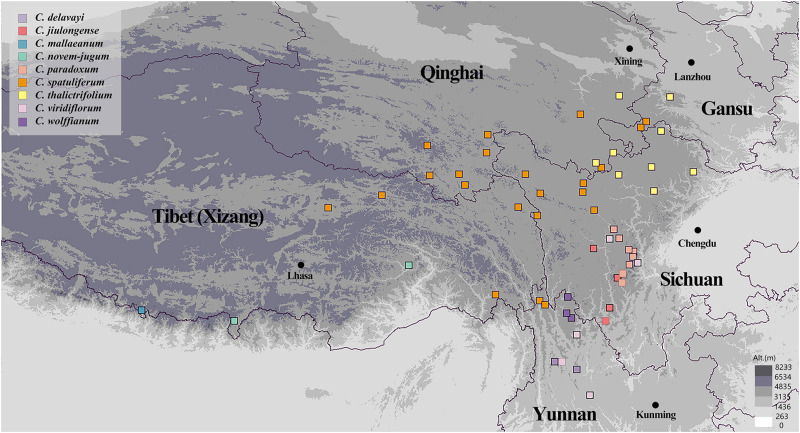
Sampling locations of *Chamaesium*.

We collected 525 individuals belonging to nine species of *Chamaesium* between 2015 and 2019. These individuals were from 56 populations at elevations above 3,000 m in Tibet (Xizang), Sichuan, Yunnan, Qinghai and Gansu provinces (Figure 1 and Supplementary Table 1). Sampling localities covered almost the entire distribution of the nine species. Six to 15 individuals were randomly sampled from each population with at least 20 m between the collected samples. Fresh leaves were collected and dried immediately with silica gel. Voucher specimens were deposited at Sichuan University Herbarium (SZ). *Bupleurum boissieuanum*, *Bupleurum falcatum*, and *Sanicula chinensis* were confirmed to have closely affinity with *Chamaesium*, so they were chosen as outgroups based on previous studies (Zhou et al., 2009; Guo et al., 2018).

### DNA Extraction, PCR Amplification, and Sequencing

Total genomic DNA was extracted from dried leaf tissue using a modified cetyltrimethylammonium bromide (CTAB) protocol. We amplified and sequenced ITS (ITS1–5.8sRNA–ITS2) (White et al., 1990) and three chloroplast fragments (*trnT-trnL*, *trnQ-rps16*, *rpl16*) (Shaw et al., 2005, 2007; Danderson et al., 2017). Polymerase chain reactions (PCRs) were performed in a 30 μL volume with 3 μL plant total DNA, 1.5 μL forward primer, 1.5 μL reverse primer and 15 μL volume 2×Taq MasterMix (cwbio, Beijing, China). All sequences in this study were deposited in GenBank with accession numbers MT678849–MT678858, MT723793–MT723822, MT827288–MT827802, and MT850316–MT851860.

### Phylogeny Reconstruction

All the DNA sequences were edited by SeqMan (DNAstar package; DNAStar Inc., Madison, WI, United States) to obtain consensus sequences. We used program MAFFT v.7.369b (Katoh and Standley, 2013) to align for subsequent manual adjustments. Haplotypes were identified and distinguished using DNAsp version 5.0 (Librado and Rozas, 2009). Gaps were treated as missing data during the tree searches. The phylogeny reconstruction based on haplotype was performed using Bayesian inference (BI) and maximum likelihood (ML). The BI and ML analyses were conducted with MrBayes version 3.2 (Ronquist et al., 2012) and IQ-TREE 1.2.12 (Nguyen et al., 2015). The best model for ITS and cpDNA were GTR + G (BI) or GTR + I + G (ML) inferred by ModelFinder (Kalyaanamoorthy et al., 2017) based on the Akaike information criterion (AIC), respectively. BI was performed using a Markov chain Monte Carlo (MCMC) method, as implemented in MrBayes, v.3.2.6 (Ronquist et al., 2012). We set the number of generations to 10 million, sampling every 1,000 generations, discarding 25% of the trees as burn-in in BI. All runs were inspected to check that the average standard deviation of split frequencies was <0.1 (Ronquist et al., 2012). Besides, we ran IQ-TREE on ITS and cpDNA with the ultrafast bootstrap feature, “-bb 10000” (Hoang et al., 2018), using the ‘‘-alrt 1000’’ option to assess branch supports. The convergence of MCMC inference was evaluated using Tracer v.1.7.1^1^. Finally, ML bootstrap support (ML BS) and BI posterior probabilities (BI PP) were presented at the nodes.

### Divergence Time Estimate

There are no *Chamaesium* fossil records, yet there are relatively consistent results of the crown age of *Chamaesium* based on cpDNA and ITS (Calvino et al., 2016; Wen et al., 2020). Therefore, we conducted divergence time estimation using BEAST (Drummond and Rambaut, 2007) based on calibration points from previous studies (Calvino et al., 2016; Yu et al., 2020). BEAUti was used to set criteria for analyses where we used a GTR + G substitution model selected by ModelFinder, an uncorrelated relaxed clock (Drummond et al., 2006) and a Yule process. For the ITS dataset, we applied normal priors with means of 49.78 (±3 st. dev.) and 44.88 (±5) for *Chamaesium* and *Bupleurum* crown ages, respectively. For the cpDNA dataset, the crown age of the *Chamaesium* was set to 51.4 Ma with a normally distributed standard deviation of 8. MCMC analyses were run for ten million generations with parameters sampled every 10,000 generations after discarding the first 20% of generations as burn-in. The convergence of the stationary distribution was accessed by ESS values (>200) using the Tracer v.1.7.1 (see text footnote 1). Maximum clade credibility (MCC) trees were produced with TreeAnnotator 1.8.4 in BEAST and were visualized in FigTree v1.4.4^2^.

### Ancestral Area Reconstruction

Ancestral area reconstruction is crucial to understand the biogeographic history of plant taxa by inferring origin area, developing routes and dispersal/vicariance/extinction events. We implemented a reconstruction using RASP version 4.0^3^ (Yu et al., 2020) based on Bayesian Binary Method (BBM) using the cpDNA and nrDNA datasets, respectively. We used four regions based on geographic distribution and floral composition throughout HHM and QTP regions (Wu et al., 2011) A: southern Himalayas, B: Tangut region, C: eastern Himalayas, D: Hengduan Mountains region. F81 + G rate model was applied in the BBM analysis and was run for 2 × 10^7^ generations using 1 cold chain and 9 hot Markov chains with temperature increments of 0.1.

### Population Genetics, Phylogeographic Analyses, and Demographic History

Haplotype diversity (*H*_*d*_) (Nei and Tajima, 1981) and nucleotide diversity (π) (Nei and Li, 1979) for each population were calculated using DNAsp v5.1 (Librado and Rozas, 2009) to verify the degrees and patterns of diversity. PERMUT (Pons and Petit, 1996) was used to access the total diversity (*H*_*T*_), within-population diversity (*H*_*S*_) and population differentiation indices (*G*_*ST*_ and *N*_*ST*_) (Grivet and Petit, 2002). We also used a U-statistic to test the phylogeographic structure by comparing *G*_*ST*_ and *N*_*ST*_, which can indicate the presence of phylogeographic structure. In addition, we performed analyses of molecular variance (AMOVA) with 1,000 permutations using ARLEQUIN version 3.5 (Excoffier and Lischer, 2010) to detect the genetic variation among species F_SC_, among populations within species F_ST_, and within population F_CT_. Neutrality test (Fu’s *Fs*; Tajima’s *D*) (Tajima, 1989) and mismatch distribution analysis (MDA) (Schneider and Excoffier, 1999) were conducted to test whether there was potential population expansion in *Chamaesium*. The smoothness of observed mismatch distribution was detected by the sum of squared deviations (SSD) between observed and expected mismatch distributions and Harpending’s raggedness index (*H*rag) (Harpending, 1994). Additionally, PopART 1.7 (Polzin and Daneshmand, 2003) was used to construct TCS networks among the haplotypes within *Chamaesium*.

### Species Distribution Modeling

We used MAXENT 3.3.3K (Phillips et al., 2006) to predict the distribution of *Chamaesium* during four time periods: Last Interglacial (LIG), Last Glacial Maximum (LGM), and the present and future. A total of 208 distribution sites acquired from field investigations and online herbarium records (E, K, KUN, NAS, P, PE, SZ) were used in analyses. Nineteen bioclimatic environment variables (Hijmans et al., 2005) from four time periods (Last Interglacial, Last Glacial Maximum, present, future) were downloaded from the WorldClim dataset at 2.5 min resolution and employed in analyses. We used 19 bioclimatic variables involving altitude, temperature and precipitation (WorldClim) with significant effects within *Chamaesium* to detect changes in distribution between the four time periods. We also tested the area under the “Receiver Operating Characteristic (ROC) curve” (AUC) (Peterson et al., 2008; Elith and Leathwick, 2009) to observe the accuracy of each model prediction. Good model performance was assessed by AUC values above 0.7 (Fielding and Bell, 1997).

## Results

### Genetic Diversity and Structure

**FIGURE 2:**
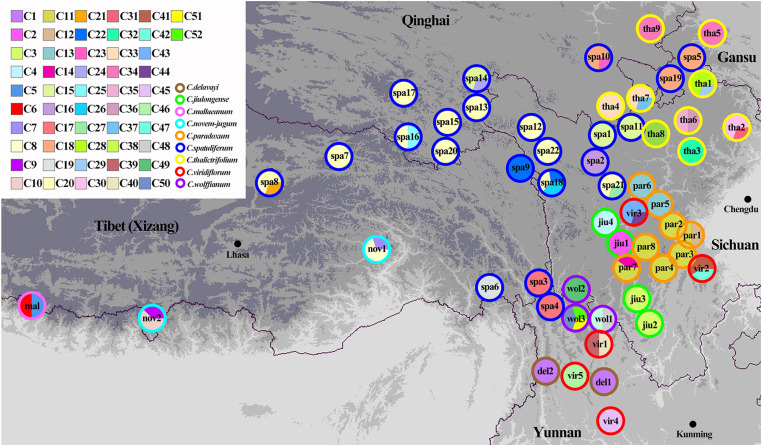
Geographic distribution of chloroplast haplotypes for *Chamaesium*. Each circle represents a population and the colored outlines of the circles distinguish the nine species. Each color represents a haplotype.

**FIGURE 3:**
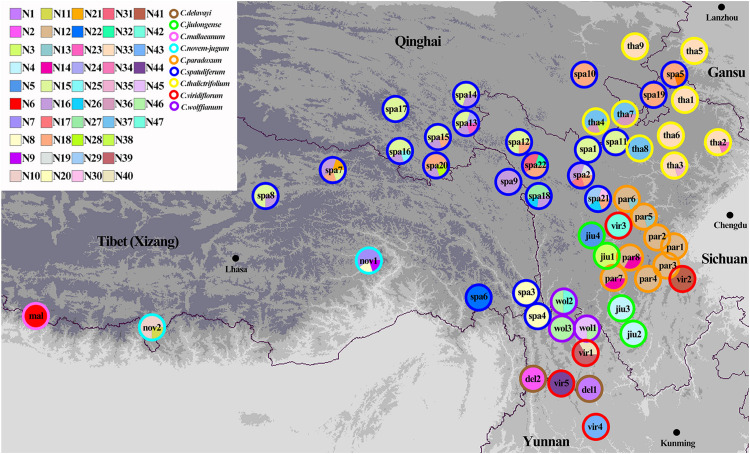
Geographic distribution of ITS haplotypes for *Chamaesium*. Each circle represents a population and the colored outlines of the circles distinguish the nine species. Each color represents a haplotype.

**FIGURE 4:**
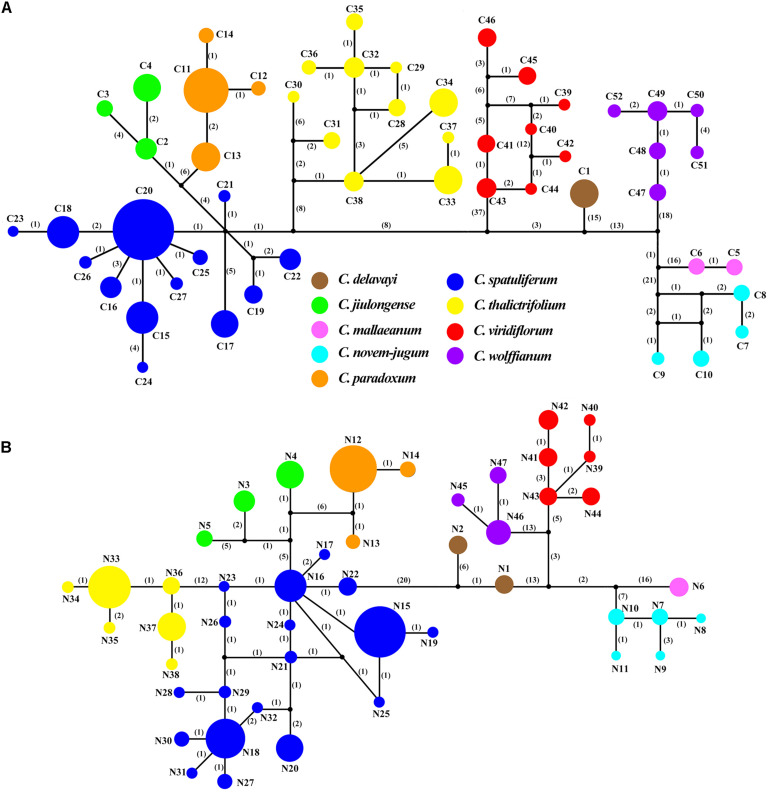
TCS networks for **(A)** 52 chloroplast haplotypes **(B)** 47 ITS haplotypes in *Chamaesium*. Each color represents a species. The size of circles in the network corresponds to the frequency of each haplotype. Numbers on the branches indicate the number of mutations between haplotypes. Small solid black circles denoted hypothetic unsampled or extinct ancestral haplotypes.

**FIGURE 5:**
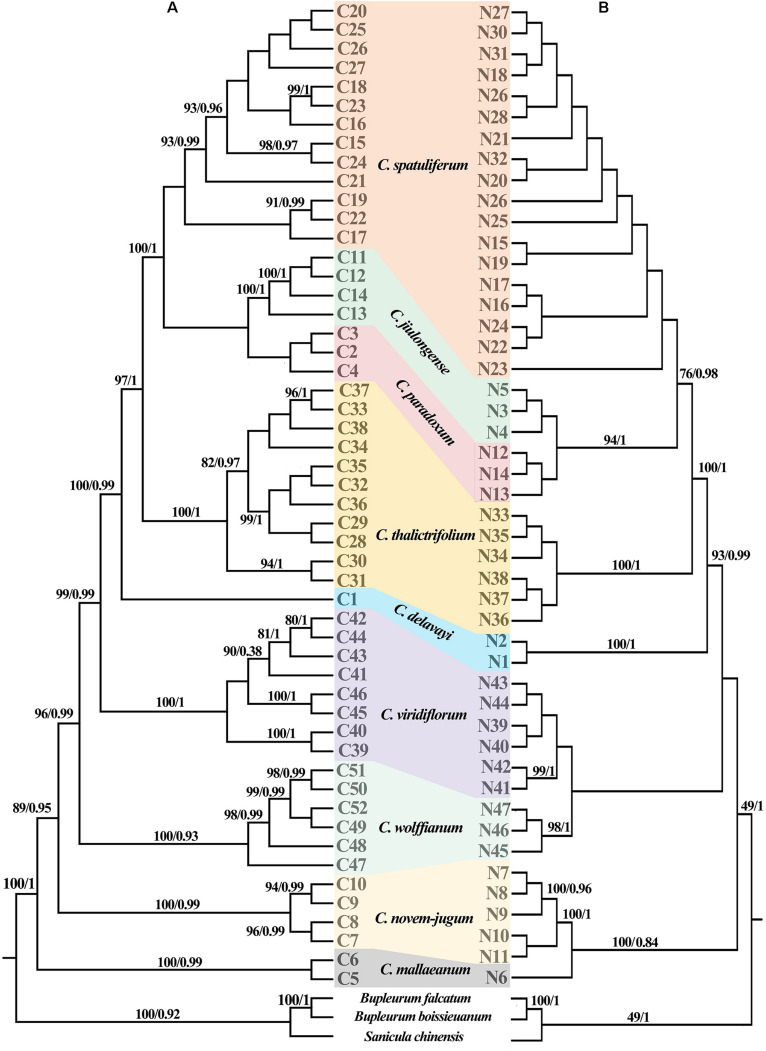
Phylogenetic relationship of *Chamaesium* recovered from the **(A)** chloroplast haplotypes and **(B)** ITS haplotypes. The maximum likelihood (ML) tree was only shown for the similar topologies with bayesian inference (BI) tree. Posterior probabilities (PP) and bootstrap values (BS) are shown above the branches, separated by backslash (/).

Three chloroplast fragments (*trnT-trnL*, *trnQ-rps16*, *rpl16*) and ITS were used to analyze 525 individuals from 56 populations of the nine *Chamaesium* species. The cpDNA and ITS haplotype frequencies of each population are listed in Supplementary Table 2, and the geographical distributions of haplotypes are shown in Figures 2, 3. The total length of the aligned sequences of cpDNA was 1,776 bp containing 201 polymorphic sites. We detected 52 chloroplast haplotypes (C1–C52), which were species-specific as none were shared by any two species (Figure 4A). 64.29% of the populations from each studied species were fixed for a single haplotype, and more than 32.14% of the populations have two haplotypes. In *C. delavayi*, only one haplotype (C1) was shared by two populations. A population with three haplotypes only occurred in *C. spatuliferum* (C20, C22, C26) and *C. wolffianum* (C50, C51, C52). Besides, the sequenced ITS’s total length was 635 bp, and 106 polymorphic sites were recovered with 47 haplotypes (N1–N47). Like cpDNA haplotypes, all ITS haplotypes were species-specific in *Chamaesium* (Figure 4B), and more than 53.57% of the populations from each studied species were fixed for a single haplotype and 26.78% of the populations had two haplotypes. A population with three or four haplotypes only occurred in *C. spatuliferum*. Simultaneously, all species form a monophyletic group in the haplotype network and the phylogenetic tree (Figure 5).

The haplotype diversity (*H*_*d*_) of cpDNA ranged from 0.000 to 0.733, and nucleotide diversity (π) ranged from 0.000 to 0.002 across all species (Supplementary Table 2). Total gene diversity (*H*_*T*_) value was higher than average gene diversity within populations (*H*_*S*_) at the level of genus. Additionally, the number of substitution types (*N*_*ST*_) was higher than interpopulation differentiation (*G*_*ST*_), which indicated that a significant phylogeographic structure existed in *Chamaesium* (Table 1). The haplotype diversity (*H*_*d*_) of ITS ranged from 0.000 to 0.800, and nucleotide diversity (π) ranged from 0.000 to 0.005 across all species (Supplementary Table 2). Like cpDNA haplotypes, the total gene diversity (*H*_*T*_) value was higher than the average gene diversity within populations (*H*_*S*_), whether at the level of species or genus. Total *N*_*ST*_ was significantly higher than *G*_*ST*_, indicating significant phylogeographical structure in *Chamaesium* (Table 1).

**TABLE 1:** Genetic diversity and genetic differentiation of nine species within *Chamaesium* based on cpDNA and ITS.

Species	*H*_*S*_	*H*_*T*_	*G*_*ST*_	*N*_*ST*_	*N*m
**cpDNA**					
*C. delavayi*	–	–	–	–	–
*C. jiulongense*	0	0.833 (0.144)	1 (NC)	1 (NC)	0
*C. mallaeanum*	–	–	–	–	–
*C. novem-jugum*	–	–	–	–	0.07
*C. paradoxum*	0.136 (0.089)	0.582 (0.128)	0.766 (0.149)	0.874 (0.090)	0.04
*C. spatuliferum*	0.141 (0.052)	0.820 (0.064)	0.827 (0.062)	0.899 (0.054)	0.03
*C. thalictrifolium*	0.217 (0.086)	0.953 (0.044)	0.772 (0.087)	0.894 (0.056)	0.03
*C. viridiflorum*	0.313 (0.130)	1.000 (0.045)	0.687 (0.131)	0.935 (0.026)	0.02
*C. wolffianum*	0.513 (0.141)	1.000 (0.086)	0.487 (0.148)	0.483 (0.053)	0.27
Total	0.195 (0.009)	0.794 (0.042)	0.795 (0.009)	0.985 (0.008)	0.000
**nrDNA**					
*C. delavayi*	–	–	–	–	0
*C. jiulongense*	0	–	1 (NC)	1 (NC)	0
*C. mallaeanum*	–	–	–	–	–
*C. novem-jugum*	–	–	–	–	0.21
*C. paradoxum*	0.218 (0.085)	0.260 (0.090)	0.163 (NC)	0.166 (NC)	1.25
*C. spatuliferum*	0.357 (0.071)	0.834 (0.041)	0.572 (0.085)	0.707 (0.074)	0.1
*C. thalictrifolium*	0.207 (0.086)	0.592 (0.134)	0.650 (0.102)	0.765 (0.076)	0.08
*C. viridiflorum*	0.107 (0.107)	1.000 (0.036)	0.893 (0.107)	0.969 (0.031)	0.1
*C. wolffianum*	0.368 (0.185)	0.633 (0.165)	0.419 (0.144)	0.498 (NC)	0.25
Total	0.265 (0.008)	0.718 (0.040)	0.720 (0.008)	0.970 (0.007)	0.01

cpDNA AMOVA detected that the main genetic variation of most species (47.4–100%) occurred among populations (Table 2). In addition, the AMOVA of ITS data indicated that similar genetic differentiation patterns to those based on the cpDNA, that 16.5–100% of the overall variation was distributed among populations (Table 2).

**TABLE 2:** Analysis of molecular variance (AMOVA) of nine species within *Chamaesium* based on cpDNA and ITS.

Species	Source of variation	*d.f.*	*SS*	*VC*	*PV*(%)	Fixation indices
**cpDNA**						
All species	Among species	8	6202.82	14.950	88.56	F_SC_:0.884
	Among populations within species	47	761.972	1.707	10.11	F_ST_:0.987
	Within population	469	105.098	0.224	1.33	F_CT_:0.886
*C. delavayi*	Among populations	–	–	–	–	–
	Within population	–	–	–	–	–
*C. jiulongense*	Among populations	3	36.706	1.455	100	F_ST_:1.000
	Within population	30	0	0	0	
*C. mallaeanum*	Among populations	–	–	–	–	–
	Within population	–	–	–	–	–
*C. novem-jugum*	Among populations	1	21.564	2.357	79.33	F_ST_:0.793
	Within population	16	9.825	0.614	20.67	
*C. paradoxum*	Among populations	7	33.318	0.481	87.3	F_ST_:0.873
	Within population	70	4.9	0.07	12.7	
*C. spatuliferum*	Among populations	21	292.646	1.435	88.65	F_ST_:0.887
	Within population	189	34.7	0.184	11.35	
*C. thalictrifolium*	Among populations	8	227.667	2.943	88.97	F_ST_:0.890
	Within population	77	28.1	0.365	11.03	
*C. viridiflorum*	Among populations	4	189.525	5.935	93.39	F_ST_:0.933
	Within population	35	14.7	0.42	6.61	
*C. wolffianum*	Among populations	2	15.111	0.727	47.38	F_ST_:0.474
	Within population	25	20.175	0.807	52.62	
All samples	Among populations	55	6964.792	13.487	98.37	F_ST_:0.984
	Within population	469	105.098	0.224	1.63	
**nrDNA**						
All species	Among species	8	3937.085	9.592	88.25	F_SC_:0.773
	Among populations within species	47	448.599	0.988	9.09	F_ST_:0.973
	Within population	469	135.706	0.289	2.66	F_CT_:0.882
*C. delavayi*	Among populations	1	35	3.5	100	F_ST_:1.000
	Within population	18	0	0	0	
*C. jiulongense*	Among populations	3	64.235	2.545	100	F_ST_:1.000
	Within population	30	0	0	0	
*C. mallaeanum*	Among populations	–	–	–	–	–
	Within population	–	–	–	–	–
*C. novem-jugum*	Among populations	1	5.433	0.556	52.95	F_ST_:0.530
	Within population	16	7.9	0.494	47.05	
*C. paradoxum*	Among populations	7	3.162	0.031	16.51	F_ST_:0.165
	Within population	70	10.8	0.154	83.49	
*C. spatuliferum*	Among populations	21	248.777	1.801	68.86	F_ST_:0.689
	Within population	189	100.873	0.534	31.14	
*C. thalictrifolium*	Among populations	8	32.158	0.407	75.08	F_ST_:0.751
	Within population	77	10.4	0.135	24.92	
*C. viridiflorum*	Among populations	4	56.092	1.767	97.89	F_ST_:0.979
	Within population	35	1.333	0.038	2.11	
*C. wolffianum*	Among populations	2	3.743	0.183	50.92	F_ST_:0.509
	Within population	25	4.4	0.176	49.08	
All samples	Among populations	55	4421.684	8.547	96.73	F_ST_:0.967
	Within population	469	135.706	0.289	3.27	

*F_SC_, differentiation among species; F_ST_, differentiation among populations within species; F_CT_, differentiation within population.*

### Phylogeny Reconstruction and Divergence Time Estimation

The ML and BI topologies of cpDNA and ITS haplotype phylogenetic trees were partly inconsistent (Figure 5). Inconsistencies between the cpDNA and ITS trees occurred regarding the phylogenetic relationships of *Chamaesium mallaeanum*, *C. novem-jugum*, *C. wolffianum*, and *C. viridiflorum*. *Chamaesium mallaeanum* had a close affinity to *C. novem-jugum*, and *C. wolffianum* was sister to *C. viridiflorum* in the ITS tree. However, these four species formed their own individual branch in the cpDNA tree. Overall, no haplotypes were shared between the nine *Chamaesium* species, and each species formed its own individual branch in the ITS and cpDNA trees.

**FIGURE 6:**
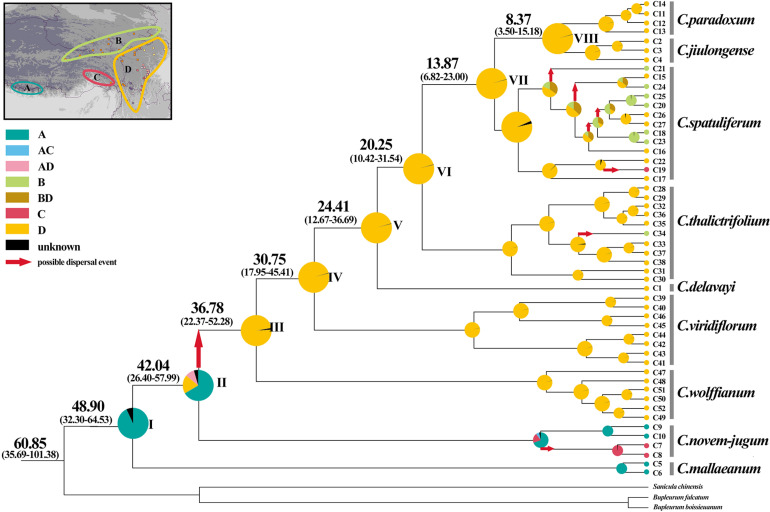
Ancestral area reconstructions based on cpDNA data set within *Chamaesium*. The map shows the four geographical distribution areas of *Chamaesium*: **(A)** southern Himalayas, **(B)** Tangut region, **(C)** eastern Himalayas, **(D)** Hengduan Mountains region. Pie charts show proportions of the ancestral ranges. The divergence time (in Ma) of the main nodes based on the cpDNA data set. Numbers in the brackets show the 95% HPD of divergence time (in Ma) of the main nodes.

**FIGURE 7:**
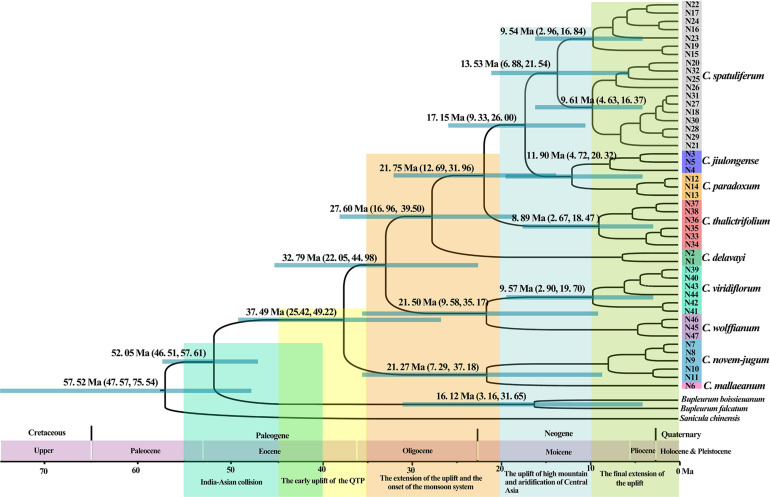
Phylogenetic chronograms of *Chamaesium* based on ITS. Green bars and numbers above represent 95% highest posterior density (95% HPDs) for each node. Geological events related to the Qinghai-Tibet Plateau are shown below the geological time scale axis, with large rectangular color blocks.

According to the beast-derived age estimate based on cpDNA (Figure 6), the crown age of *Chamaesium* was dated to be 60.85 Ma (95% HPD: 35.69–101.38 Ma). The divergence times of *C. mallaeanum* and *C. novem-jugum* were estimated at 48.90 Ma (95%HPD: 32.30–64.53 Ma) and 42.04 Ma (95%HPD: 26.40–57.99 Ma). *C. wolffianum*, *C. viridiflorum*, and *C. delavayi* branched off around 36.78 Ma (95% HPD: 22.37–52.28 Ma), 30.75 Ma (95% HPD: 17.95–45.41 Ma) and 24.41 Ma (95% HPD: 12.67–36.69 Ma), respectively. The divergence of *C. thalictrifolium*, *C. spatuliferum*, and *C. paradoxum* occurred at 20.25 Ma (95% HPD: 10.42–31.54 Ma), 13.87 Ma (95% HPD: 6.82–23.00 Ma), and 8.37 Ma (95% HPD: 3.50–15.18 Ma), respectively. The divergence times estimated from ITS (Figure 7) indicated that the crown age of *Chamaesium* was dated to be 52.05 Ma (95% HPD: 46.51–57.61 Ma). The ancestor of *C. mallaeanum* and *C. novem-jugum* separated about 37.49 Ma (95% HPD: 25.42–49.22). The ancestor of *C. wolffianum* and *C. viridiflorum* separated around 32.79 Ma (95% HPD: 22.05–44.98). Furthermore, the divergence times of *C. delavayi*, *C. thalictrifolium*, *C. spatuliferum*, and *C. paradoxum* were estimated at 27.60 Ma (95% HPD: 16.96–39.50 Ma), 21.75 Ma (95% HPD: 12.69–31.96 Ma), 17.15 Ma (95% HPD: 9.33–26.00 Ma), and 11.90 Ma (95% HPD: 4.72–20.32 Ma), respectively. The results of divergence time based on cpDNA and ITS were similar. Since the molecular markers of chloroplast are closer to neutral and have matrilineal genetic stability, which is widely used in the process of divergence time estimation and biogeographic deduction, the results obtained cpDNA were selected for the subsequent elaboration of the differentiation history of *Chamaesium*.

### Ancestral Area Reconstruction

The BBM analysis of ancestral distribution (Figure 6) areas was used to indicate the ancient distribution of *Chamaesium* in the southern Himalayas based on cpDNA data (node I). Several ancestral populations moved eastwards to the eastern Himalayas and continued eastward, diversifying in the HHM region during the early Oligocene. One vicariance and two dispersal events were identified within populations from the eastern Himalayas to the HHM region (node II). Furthermore, the BBM analyses identified several dispersal events within *C. novem-jugum*, *C. thalictrifolium*, and *C. spatuliferum*. For example, *C. spatuliferum* populations separated and one group colonized the Tangut region or eastern Himalayas from HHM region, while the other continued diversifying in the HHM region.

### Demographic History and Species Distribution Modeling

**FIGURE 8:**
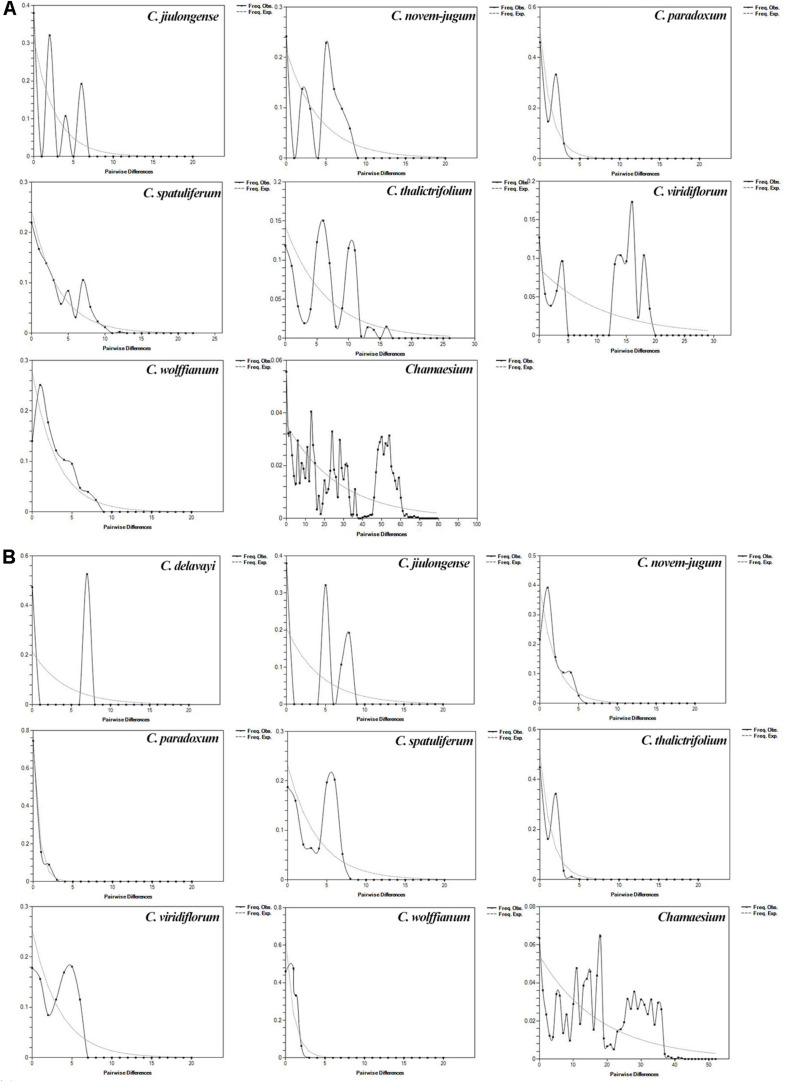
Mismatch distribution analysis for ITS **(A)** and cpDNA **(B)**. The line represents the distributions of an expected population expansion, the dashed line show observed (Obs) values.

Based on cpDNA and ITS data, we performed mismatch distribution analysis and neutrality test to determine the demographic history of nine species. Values of Tajima’s *D* and Fu’s *Fs* were non-significant for all species in the neutrality test (Table 3). The mismatch distributions for our study species were multimodal and/or very ragged, which indicates populations are stable and not shrinking (Figure 8).

**TABLE 3:** Mismatch distribution analysis and neutrality tests within *Chamaesium* based on cpDNA and ITS.

Species	Tajima’s *D* (*p*)	Fu’s *Fs* (*p*)	SSD (*p*)	*H*rag (*p*)
**cpDNA**				
*C. delavayi*	–	–	–	–
*C. jiulongense*	0	0	0	0
*C. mallaeanum*	–	–	–	–
*C. novem-jugum*	1.317 (0.905)	2.520 (0.877)	0.224 (0.125)	0.755 (0.085)
*C. paradoxum*	0.346 (0.990)	0.266 (N.A.)	0.008 (0.038)	0.076 (0.033)
*C. spatuliferum*	0.284 (0.968)	0.538 (N.A.)	0.040 (0.042)	0.128 (0.095)
*C. thalictrifolium*	0.543 (0.960)	1.206 (N.A.)	0.059 (0.063)	0.220 (0.109)
*C. viridiflorum*	0.863 (0.954)	1.563 (N.A.)	0.147 (0.050)	0.470 (0.060)
*C. wolffianum*	0.597 (0.696)	1.573 (0.567)	0.199 (0.080)	0.451 (0.183)
All	0.446 (0.969)	0.744 (N.A.)	0.052 (0.055)	0.174 (0.092)
**nrDNA**				
*C. delavayi*	0	0	0	0
*C. jiulongense*	0	0	0	0
*C. mallaeanum*	–	–	–	–
*C. novem-jugum*	1.780 (0.667)	0.865 (0.603)	0.373 (0.304)	0.007 (0.255)
*C. paradoxum*	0.291 (0.876)	0.616 (N.A.)	0.038 (0.076)	0.234 (0.141)
*C. spatuliferum*	0.234 (0.822)	0.851 (N.A.)	0.047 (0.144)	0.184 (0.217)
*C. thalictrifolium*	0.151 (0.879)	0.313 (N.A.)	0.022 (0.096)	0.155 (0.137)
*C. viridiflorum*	0.170 (0.972)	0.125 (N.A.)	0.006 (0.054)	0.058 (0.072)
*C. wolffianum*	0.916 (0.955)	0.665 (N.A.)	0.024 (0.080)	0.212 (0.067)
All	0.231 (0.882)	0.550 (N.A.)	0.039 (0.101)	0.154 (0.173)

**FIGURE 9:**
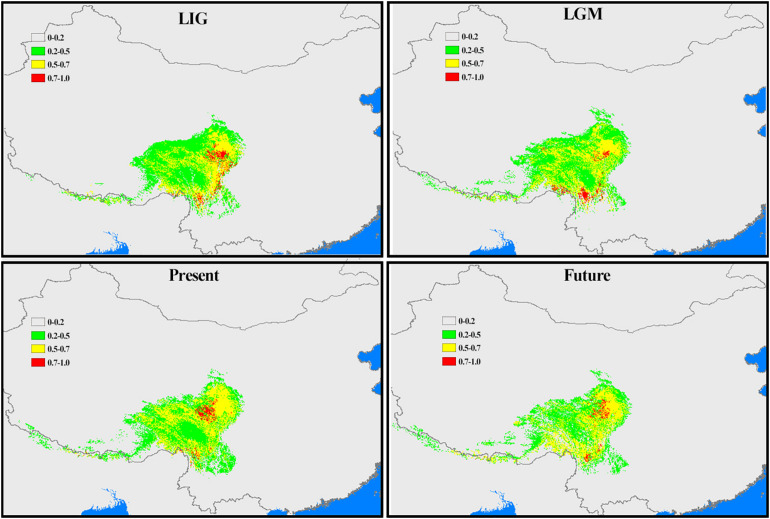
Potential distribution ranges within *Chamaesium* during LIG, LGM, present and future were simulated by using bioclimatic variables. Colors represent species climate adaptability.

Considering *Chamaesium* and climate scenarios (the LIG, the LGM, the present and the future), the area under the AUC value for the potential climatically suitable areas of *Chamaesium* was high (>0.95), indicating the highest predictive capacity. The distribution ranges predicted for *Chamaesium* were consistent with the actual geographic distributions. This modeling (Figure 9) showed that the overall simulated distribution range of *Chamaesium* in LIG, LGM, the present and the future is not very different. The main difference lies in the change of the optimal distribution range (red) of *Chamaesium*. The climate decreased sharply from LIG to LGM, and the optimal distribution range of *Chamaesium* changed from the northern to the southern part of Hengduan Mountain. From LGM to the present, the main optimum distribution range changed from the southern to the northern part of the Hengduan Mountains. The optimal distribution area in the future is obviously reduced compared with that in the present.

## Discussion

### Genetic Diversity and Structure

Genetic diversity is the product of the long-term evolution of species, which is caused by many factors and is one of the prerequisites for survival, adaptation and evolution of species (Soltis and Soltis, 1991). The higher the level of genetic diversity of a species, the stronger its adaptability to environmental changes, the easier it is to expand to other distribution ranges or develop new living environments. On the contrary, when a species lacks genetic diversity, it will be unable to adapt to the complex and changeable environment, leading to the extinction of species (Avise and Hamrick, 1996). Petit et al. (2005) has integrated data related to the genetic diversity of a variety of plant groups, and the results show that the average genetic diversity of angiosperms is 0.67 based on chloroplast data analysis. In this study, we found that *Chamaesium* showed a high level of total genetic diversity based on the two datasets (Table 1), which was significantly higher than the average value of angiosperm genetic diversity obtained by Petit et al., and the high level of genetic diversity of *Chamaesium* could be attributed to the following reasons: (1) *Chamaesium* originated around the beginning of Paleogene. During its long evolutionary history, *Chamaesium* has experienced many geological events, climate changes and accumulated a lot of genetic variation. (2) *Chamaesium* is commonly distributed at an altitude of more than 3,000 m in the HHM, where the topography is very complex. Habitat fragmentation caused by the unique “island sky” prompts the differentiation among populations and increases the probability of genetic drift within the population, which will accumulate abundant genetic variation. (3) The reproductive mode of *Chamaesium* is sexual reproduction, which makes the species not only to maintain genetic stability but also enrich genetic variability. Generally speaking, a high level of genetic diversity plays a crucial role in maintaining both the biodiversity of plant groups and the dynamic balance of population (Hughes et al., 2008; Jump et al., 2009). Similar results have been found in other alpine plants distributed on the Tibetan Plateau, such as *Pedicularis longensis* (cpDNA: *H*_*T*_ = 0.77), *Eriophyton wallichii* (cpDNA: *H*_*T*_ = 0.983; ITS: *H*_*T*_ = 0.953), *Thalictrum squamiferum* (cpDNA: *H*_*T*_ = 0.973), *Paraquilegia microphylla* (cpDNA: *H*_*T*_ = 0.984; G3pdh: *H*_*T*_ = 0.977), *Chionocharis hookeri* (cpDNA: *H*_*T*_ = 0.935; ITS: *H*_*T*_ = 0.944), *Notopterygium incisum* (cpDNA: *H*_*T*_ = 0.939; ITS: *H*_*T*_ = 0.725) (Yang et al., 2008; Wang et al., 2011; Shahzad et al., 2017).

In addition, AMOVA was performed based on two datasets. The results showed (Table 2) that if *Chamaesium* was taken as a whole, the genetic variation among 56 populations was large (ITS: 96.73%; cpDNA: 98.37%) and the genetic variation within the population was relatively small (ITS: 3.27%; cpDNA: 1.63%), which may be related to the following reasons: (1) Field studies have shown that the seed/pollen dispersal capacity of *Chamaesium* is very limited, and the gene flow levels of populations based on two types of datasets are very low. Most of the inter-population differentiation may be due to limited gene flow. Similar results were found in *Quercus* sect. *Heterobalanus*, *Allium* section *Sikkimensia*, *Angelica nitida*, *Bupleurum longiradiatum*, and *Thalictrum squamiferum* (Zhang and He, 2013; Zhao et al., 2013; Luo et al., 2016; Meng et al., 2017; Xie et al., 2018). (2) It is found that the genetic differentiation of many organisms in the HHM is mainly driven by complex geological activities and diverse climate changes, such as habitat fragmentation can decrease gene flow and increase genetic variation among populations. Therefore, the higher genetic differentiation among *Chamaesium* populations may be related to the drastic changes in the external environment in this region. However, the AMOVA results of *C. paradoxum* based on ITS data indicated that the genetic variation was mainly within populations (83.49%), while the AMOVA results of *C. wolffianum* based on the two datasets indicated that the probability of genetic variation between populations (ITS: 50.92%; cpDNA:47.38%) and within populations (ITS:49.08%; cpDNA:52.62%) was very similar. Except for *C. mallaeanum* and *C. delavayi*, the genetic variation of other species occurred mainly between populations. The reasons for this genetic structure inconsistency are speculated as follows: (1) Three haplotypes (N12, N13, N14) were detected in the eight populations of *C. paradoxum* based on ITS data, of which N12 was the haplotype shared by the eight populations, thus reducing the genetic variation among the populations. (2) Six haplotypes (C47–C52) were detected in three populations of *C. wolffianum* based on cpDNA. These haplotypes were all unique and existed within and between populations of *C. wolffianum*, so the proportion of genetic variation was very close between and within populations.

Both ITS and cpDNA datasets showed a high level of haplotype diversity. We also constructed haplotype TCS networks of *Chamaesium* using PopART and drew geographic distribution maps of haplotypes (Figure 4). The results showed that there were no shared haplotypes among the nine *Chamaesium* species, and most (cpDNA/nDNA) haplotypes were restricted to single sites or neighboring populations. Therefore, we speculate that due to a combination of extrinsic and species-specific factors, these high-altitude populations remained isolated and confined within a mountainous area, not only during the glacial period but also during the interglacial period. This is mainly because: (1) The Himalaya-Hengduan Mountain region has complex topographic features, especially in the Hengduan Mountain region, where many high mountains above 4,000 m are separated by valleys. (2) Limited pollen/seed dispersal capacity of *Chamaesium*. (3) These alpine plants are not adapted to lowland conditions. Nevertheless, different haplotypes were found in a large number of populations, both based on ITS and cpDNA datasets, which still requires further investigation.

### Origin and Diversification of *Chamaesium*

In the long history of the evolution of the earth, every geological activity and climate change will bring unprecedented effects on organisms, which is also a hot topic of research at present. In the present study, all *Chamaesium* populations we collected were mainly distributed in four regions: A: southern Himalayas, B: Tangut region, C: eastern Himalayas, D: Hengduan Mountains region. We estimate the time of the origin of *Chamaesium* and the differentiation of nine species and reconstruct the ancestral distribution of *Chamaesium* (Figure 6). The results show that the ancestral group of *Chamaesium* originated in the southern Himalayan region at the beginning of the Paleogene (60.85 Ma, 95% HPD: 35.69–101.38 Ma). And the most widely accepted theory is that the uplift of the QTP resulting from the collision of the Indian plate with Eurasia began at about 55–50 Ma, which also led to dramatic changes in the topography of this region. Great habitat changes have also contributed to the formation and differentiation of species, such as *Gentiana* (Motley, 2003; Favre et al., 2016) and *Saxifraga* (Ebersbach et al., 2017). Therefore, we speculate that the origin of *Chamaesium* is closely related to the early uplift of the QTP caused by the collision. The BEAST analysis showed that *C. mallaeanum* and *C. novem-jugum* diverged in succession in the early and middle Eocene. BBM analysis showed that some ancestral populations migrated eastward into the eastern Himalayas, during which time the QTP continued to rise from south to north.

During the Oligocene (36.5–23 Ma), the uplift progressed particularly northward and southward, causing the extension of the QTP. Due to its considerable size and elevation, the QTP and its adjacent mountains progressively acted as an orographic barrier to the Asian atmospheric circulation, which became a direct cause of the formation of the monsoon climate. BEAST analysis showed that *C. wolffianum* separated during the early Oligocene (36.78 Ma, 95% HPD: 22.37–52.28 Ma) and the dispersal event at node II (Figure 6) tested in BBM indicated that some populations of *Chamaesium* migrated eastward to the Hengduan Mountain region. *C. viridiflorum* and *C. delavayi* were detected that differentiated in the middle (30.75 Ma, 95% HPD: 17.95–45.41 Ma) and late Oligocene (24.41 Ma, 95% HPD: 12.67–36.69 Ma). Therefore, we infer that the colonization of the newly available climate and terrain facilitated the differentiation of several species in *Chamaesium*. Similar results have been found in other species, such as *Primulina*, *Cardiocrinum*, and *Saxifraga* (Yang et al., 2016; Ebersbach et al., 2017).

Before the late Miocene (23–10 Ma), both the Himalayas and the Tianshan Mountains experienced considerable elevation. Combined with the global temperature drop since the Miocene, drought events occurred in Central Asia. Some studies have shown that other factors, including changes in the size of global glaciers and the loss of the Tethys Sea, contributed to the Central Asian Aridification. The dramatic reduction in rainfall has led to great changes in many biological communities, such as *Cyclocarya paliurus*, *Cardiocrinum*, and *Rabdosia* (Yu et al., 2014; Yang et al., 2016). Our molecular dating analysis suggested that *C. thalictrifolium* and *C. spatuliferum* differentiated in the early (20.25 Ma, 95% HPD: 10.42–31.54 Ma) and middle (13.87 Ma, 95% HPD: 6.82–23.00 Ma) Miocene. Therefore, we speculate that the division of *Chamaesium* at this time is closely related to the drought event in Central Asia and the change of habitat topography.

In the late Miocene and later (10 Ma–present), QTP experienced further uplift and expansion, especially in the eastern margin region, which contains many mountain ranges, most notably the Hengduan biodiversity hotspot. Warm and moist air from the Indian Ocean is blocked by the Himalayas and the KailasRange, so it enters China from the Hengduan Mountains (Lopez-Pujol et al., 2011; He and Jiang, 2014; Sklenar et al., 2014; Song et al., 2016). This has brought plenty of rain to the southeastern part of QTP, which in turn has had a dramatic effect on species there, such as *Allium* section *Sikkimensia*, *Cardiocrinum*, *Dolomiaea*, *Rheum*, and *Myricaria* (Miehe et al., 2007; Sun et al., 2012; Zhang et al., 2014; Yang et al., 2016; Xie et al., 2018). BEAST analysis showed that the split of *C. paradoxum* and *C. jiulongense* occurred in the late Miocene (8.37 Ma, 95% HPD: 3.50–15.18 Ma). In addition, BBM analysis showed that there were some dispersal events in *C. thalictrifolium* and *C. spatuliferum*. We infer that complex geological activity and climate change play important roles in the differentiation and dispersal of *Chamaesium*.

### Population and Range Dynamics Within *Chamaesium*

Quaternary glaciation has always been the background event discussed in the phylogeography study. Drastic fluctuation in the climate is generated by the recurrence of glaciation, which caused large-scale migration of organisms and natural disaster avoidance for survival. At the same time, the genetic structure and distribution of the surviving organisms can change dramatically (Taberlet et al., 1998). QTP never formed a unified ice sheet during the glacial period, and the terrain in the subtropical region is complex and diverse. Even in the harsh climate conditions during the glacial period, there are still some relatively warm and humid places for organisms to survive. Such natural isolation places may also lead to the differentiation of species or promote the formation of subspecies and new species. Therefore, regions with high levels of genetic diversity, ancient haplotypes or endemic haplotypes are often speculated as the glacial refuge of organisms in the study of phylogeography (Tzedakis, 2002).

According to our study mentioned above, nine species of *Chamaesium* have separated well during the Miocene. Our maxent modeling predicted that the simulated distribution range of *Chamaesium* did not change much during LIG, LGM and present (Figure 9). Non-significant results of the neutrality test, multimodal distribution shapes of the mismatch distribution analysis (Figure 8 and Table 3) and non-star-like phylogeographical structure of haplotype (Figure 4) implied that the populations are stable and not shrinking. We suggested that the stability of the overall distribution range of *Chamaesium* may be the result of the combined effects of the characteristics of *Chamaesium* and the external environment. As a cold-tolerant species, *Chamaesium* is distributed in the Himalaya-Hengduan Mountains region, and all the plant groups grow in the mountains above 3,000 m. Many studies have shown that the absence of unified ice-sheets covering the entire QTP and bordering mountains and plateau glacier expansion was less pronounced in the QTP than in other regions of the Northern Hemisphere. Therefore, climate change caused by glaciers was not an entirely adverse factor for the flora in our study area. During Quaternary glaciation, some cold-tolerant plants with a wide range of habitats on QTP could survive in multiple refuges. We have detected high levels of genetic diversity (ITS: *H*_*d*_ = 0.8) and endemic haplotypes in populations from spa2, spa13, and spa21 (Supplementary Table 2), suggesting that Hengduan Mountain and its adjacent areas may have provided multiple glacial refuges for *Chamaesium*. Similar results have also been found in other groups, such as *Pedicularis longicornis*, *Metagentiana striata*, *Picea likiangensis*, *Potentilla glabra*, *Eriophyton wallichi*, *Primula secundiflora*, and *Rhodiola alsia* (Chen et al., 2008; Wang L.Y. et al., 2009; Yang et al., 2008; Gao et al., 2011; Wang et al., 2011). These studies suggested that the Quaternary glaciation had little effect on the survival and widespread distribution of alpine cold-tolerant plants in the subtropical regions of QTP, in contrast to the results of mountains in temperate regions that were covered by more extensive ice sheets during the Quaternary.

## Data Availability Statement

The datasets presented in this study can be found in online repositories. The names of the repository/repositories and accession number(s) can be found below: https://www.ncbi.nlm.nih.gov/genbank/, MT678849–MT678858, MT723793–MT723822, MT827288–MT827802, MT850316–MT851860.

## Author Contributions

H-YZ, X-LG, X-JH, and S-DZ conceived the idea. H-YZ and X-LG contributed to the collecting data, designed, and performed the experiment, and prepared the manuscript. H-YZ, X-LG, MP, X-JH, and S-DZ authored the drafts of the article. All authors contributed, made multiple revisions, and approved the final draft.

## Conflict of Interest

The authors declare that the research was conducted in the absence of any commercial or financial relationships that could be construed as a potential conflict of interest.

## Footnotes

1 http://beast.bio.ed.ac.uk/Tracer

2 http://tree.bio.ed.ac.uk/software/figtree/

3 http://mnh.scu.edu.cn/soft/blog/RASP/index.html
