# Supplementary material for: Effects of Mountain Uplift and Climatic Oscillations on Phylogeography and Species Divergence of Chamaesium (Apiaceae)
Source: Front Plant Sci. 2021 May 24;12:673200. doi: 10.3389/fpls.2021.673200 (PMC8183463; doi:10.3389/fpls.2021.673200)
Supplement: Supplementary file 1 [file Data_Sheet_1.docx]

Supplementary Material

**Supplementary Table 1.** Sampling information of *Chamaesium*.

| **Population Code** | **Location** | **Latitude** | **Longitude** | **Altitude(m)** | **Sample** | **Voucher Specimen** |
| --- | --- | --- | --- | --- | --- | --- |
| *Chamaesium delavayi* (del) |  |  |  |  |  |  |
| del1 | Maer Mts., Heqing, Yunnan | 26.241 | 100.1189 | 3590 | 10 | GXL16082701 |
| del2 | Xuebang Mts., Lanping, Yunnan | 26.4783 | 99.3989 | 3669 | 10 | GXL19102001 |
| *Chamaesium jiulongense* (jiu) |  |  |  |  |  |  |
| jiu1 | Haite Mts., Jiulong, Sichuan | 29.1998 | 101.4275 | 3917 | 10 | GXL16091001 |
| jiu2 | Yigongduan, Muli, Sichuan | 27.9264 | 101.6389 | 3452 | 9 | GXL18081103 |
| jiu3 | Yazuizu, Muli, Sichuan | 28.2295 | 101.1721 | 3324 | 9 | GXL18080801 |
| jiu4 | Yajiang, Sichuan | 30.1431 | 100.6525 | 4425 | 6 | GXL19090301 |
| *Chamaesium mallaeanum* (mal) |  |  |  |  |  |  |
| mal | Nielamu Town, Nielamu, Tibet | 28.1579 | 85.9823 | 3689 | 10 | GXL15090101 |
| *Chamaesium novem-jugum* (nov) |  |  |  |  |  |  |
| nov1 | Sejila Mts., Linzhi, Tibet | 29.6069 | 94.653 | 4646 | 10 | GXL15090501 |
| nov2 | Kangbu hot spring, Yadong, Tibet | 27.8074 | 88.9863 | 4227 | 8 | GXL16082301 |
| *Chamaesium paradoxum* (par) |  |  |  |  |  |  |
| par1 | Mugecuo,Kangding, Sichuan | 30.1731 | 101.871 | 3567 | 10 | GXL15092501 |
| par2 | Zheduo Mts., Kangding, Sichuan | 30.07 | 101.8042 | 4382 | 9 | GXL18081504 |
| par3 | Gaojiagou, Kangding, Sichuan | 29.872 | 101.9286 | 4129 | 10 | GXL18081403 |
| par4 | Gongga Mts., Kangding, Sichuan | 29.6194 | 101.8163 | 4856 | 10 | XYP19081803 |
| par5 | Bamei Town, Daofu, Sichuan | 30.4831 | 101.4841 | 3442 | 10 | GXL15092701 |
| par6 | Songlinkou, Daofu, Sichuan | 30.7813 | 101.3189 | 3946 | 10 | ZHY19072701 |
| par7 | Haite Mts., Jiulong, Sichuan | 29.1451 | 101.5069 | 3249 | 10 | GXL15091502 |
| par8 | Riluku, Jiulong, Sichuan | 29.208 | 101.4414 | 3634 | 9 | GXL18081601 |
| *Chamaesium spatuliferum* (spa) |  |  |  |  |  |  |
| spa1 | Seda, Sichuan | 32.269 | 100.3326 | 3890 | 10 | GXL15082701 |
| spa2 | Nibagou, Gannzi, Sichuan | 31.9681 | 100.3073 | 3849 | 10 | GXL15092801 |
| spa3 | Deqin, Yunnan | 28.4641 | 98.9169 | 3174 | 10 | GXL16083101 |
| spa4 | Baima Snow Mts., Deqin, Yunnan | 28.3342 | 99.0787 | 4337 | 9 | GXL16073001 |
| spa5 | Gahai, Luqu, Gansu | 34.2583 | 102.3678 | 3518 | 10 | GXL16082501 |
| spa6 | Chayu, Tibet | 28.6617 | 97.4668 | 3432 | 7 | GXL18081701 |
| spa7 | Suoxian, Tibet | 31.8869 | 93.7855 | 3987 | 10 | GXL15083001 |
| spa8 | Kongma, Naqu, Tibet | 31.469 | 92.0452 | 4507 | 10 | GXL15082001 |
| spa9 | Jiangda, Tibet | 31.4998 | 98.2182 | 3605 | 8 | GXL15081501 |
| spa10 | Dawu, Maqin, Qinghai | 34.4914 | 100.2202 | 3724 | 8 | GXL16072901 |
| spa11 | Banma, Qinghai | 32.7625 | 100.9147 | 3724 | 8 | GXL16071802 |
| spa12 | Jumu Vil., Shiqu, Sichuan | 32.5656 | 98.4359 | 4251 | 10 | ZHY19081301 |
| spa13 | Xiewu Town, Yushu, Qinghai | 33.8473 | 97.2133 | 4427 | 10 | ZHY19081501 |
| spa14 | Zhenqin Town, Chengduo, Qinghai | 33.2491 | 97.1752 | 4076 | 10 | ZHY19081701 |
| spa15 | Eyala, Nangqian, Qinghai | 32.5629 | 96.2847 | 4435 | 10 | ZHY19081901 |
| spa16 | Angsai Vil., Zaduo, Qinghai | 32.5145 | 95.3364 | 4219 | 10 | ZHY19082001 |
| spa17 | Duocai Vil., Zhiduo, Qinghai | 33.4832 | 95.2628 | 4815 | 10 | ZHY19082101 |
| spa18 | Baiyu, Sichuan | 31.2103 | 98.8241 | 3317 | 11 | GXL19080801 |
| spa19 | Zhongke, Maqu, Gansu | 34.0667 | 102.1818 | 4054 | 10 | ZHY19092901 |
| spa20 | Jiangxilinchang, Nangqian, Qinghai | 32.2021 | 96.4769 | 3630 | 10 | GXL19092201 |
| spa21 | Luhuo, Sichuan | 31.4004 | 100.6602 | 3265 | 10 | ZHY19092301 |
| spa22 | Queer Mts., Dege, Sichuan | 31.9405 | 98.9234 | 4899 | 10 | GXL19092601 |
| *Chamaesium thalictrifolium* (tha) |  |  |  |  |  |  |
| tha1 | Huahu, Ruoergai, Sichuan | 33.9484 | 102.8441 | 3495 | 10 | GXL15092001 |
| tha2 | Xuebaoding, Songpan, Sichuan | 32.6763 | 103.8468 | 3300 | 10 | GXL15091801 |
| tha3 | Shuajingsi, Maerkang, Sichuan | 32.0148 | 102.6164 | 3365 | 10 | GXL16091301 |
| tha4 | Qingshui River, Banma, Qinghai | 32.9326 | 100.7373 | 3504 | 10 | GXL16071801 |
| tha5 | Zuogaiduoma, Hezuo, Gansu | 35.0549 | 103.1335 | 3421 | 8 | GXL16080801 |
| tha6 | Longri Vil., Hongyuan, Sichuan | 32.7908 | 102.5443 | 3513 | 10 | GXL18081901 |
| tha7 | Jiuzhi, Qinghai | 33.2613 | 101.2939 | 4418 | 10 | ZHY19093001 |
| tha8 | Rigezha vil., Aba, Sichuan | 32.532 | 101.465 | 4220 | 8 | ZHY19100101 |
| tha9 | Xiaderi River, Zeku, Qinghai | 35.1049 | 101.4858 | 3743 | 10 | GXL19091301 |
| *Chamaesium viridiflorum* (vir) |  |  |  |  |  |  |
| vir1 | Haba Snow Mts., Zhongdian, Yunnan | 27.3633 | 100.1108 | 3517 | 6 | GXL15071501 |
| vir2 | Yajiageng, Luding, Sichuan | 29.8789 | 102.0257 | 3821 | 9 | GXL15092301 |
| vir3 | Songlinkou, Daofu, Sichuan | 30.4647 | 101.1841 | 3804 | 10 | ZHY19072801 |
| vir4 | Cang Mts., Dali, Yunnan | 25.6602 | 100.0999 | 3916 | 6 | XYP19080405 |
| vir5 | Xuebang Mts., Lanping, Yunnan | 26.4775 | 99.4982 | 4028 | 9 | GXL19102002 |
| *Chamaesium wolffianum* (wol) |  |  |  |  |  |  |
| wol1 | Shudu Lake, Zhongdian, Yunnan | 27.9029 | 99.9344 | 3638 | 10 | GXL16082001 |
| wol2 | Da Snow Mts., Zhongdian, Yunnan | 28.5737 | 99.827 | 4255 | 8 | XYP19073003 |
| wol3 | Gezan Vil., Zhongdian, Yunnan | 28.0547 | 99.7757 | 3554 | 10 | JQP19072903 |

**Supplementary Table 2**. The haplotype distribution, haplotype diversity (*H*_d_) and nucleotide diversity (π) in each population of *Chamaesium* based on cpDNA and ITS.

| **Population code** | **Haplotype (C)** | ***H*_d_（SD)** | **π (SD)×100** | **Sample** | **Haplotype (N)** | ***H*_d_（SD)** | **π (SD)×100** |
| --- | --- | --- | --- | --- | --- | --- | --- |
| *C. delavayi* |  |  |  |  |  |  |  |
| del1 | C1(10) | 0 | 0 | 10 | N1(10) | 0 | 0 |
| del2 | C1(10) | 0 | 0 | 10 | N2(10) | 0 | 0 |
| total |  |  |  | 20 |  | 0.5263 | 0.0060 |
| *C. jiulongense* |  |  |  |  |  |  |  |
| jiu1 | C2(10) | 0 | 0 | 10 | N3(10) | 0 | 0 |
| jiu2 | C3(9) | 0 | 0 | 9 | N4(9) | 0 | 0 |
| jiu3 | C3(9) | 0 | 0 | 9 | N4(9) | 0 | 0 |
| jiu4 | C4(6) | 0 | 0 | 6 | N5(6) | 0 | 0 |
| total |  | 0.62032 | 0.00125 | 34 |  | 0.62032 | 0.0063 |
| *C. mallaeanum* |  |  |  |  |  |  |  |
| mal | C5(5), C6(5) | 0.556 | 0.00031 | 10 | N6(10) | 0 | 0 |
| *C. novem-jugum* |  |  |  |  |  |  |  |
| nov1 | C7(3), C8(7) | 0.467 | 0.00053 | 10 | N7(6), N8(2), N9(2) | 0.622 | 0.002 |
| nov2 | C9(3), C10(5) | 0.536 | 0.00091 | 8 | N10(6), N11(2) | 0.43 | 0.001 |
| total |  | 0.75817 | 0.00209 | 18 |  | 0.78431 | 0.00253 |
| *C. paradoxum* |  |  |  |  |  |  |  |
| par1 | C11(5), C12(5) | 0.556 | 0.00032 | 10 | N12(10) | 0 | 0 |
| par2 | C11(9) | 0 | 0 | 9 | N12(9) | 0 | 0 |
| par3 | C11(10) | 0 | 0 | 10 | N12(10) | 0 | 0 |
| par4 | C11(10) | 0 | 0 | 10 | N12(10) | 0 | 0 |
| par5 | C13(10) | 0 | 0 | 10 | N12(8), N13(2) | 0.356 | 0.001 |
| par6 | C13(10) | 0 | 0 | 10 | N12(8), N13(2) | 0.356 | 0.001 |
| par7 | C11(6), C14(4) | 0.533 | 0.0003 | 10 | N12(6), N14(4) | 0.533 | 0.001 |
| par8 | C11(9) | 0 | 0 | 9 | N12(6), N14(3) | 0.5 | 0.001 |
| total |  | 0.53979 | 0.00056 | 78 |  | 0.25475 | 0.00059 |
| *C. spatuliferum* |  |  |  |  |  |  |  |
| spa1 | C15(10) | 0 | 0 | 10 | N15(10) | 0 | 0 |
| spa2 | C16(10) | 0 | 0 | 10 | N16(4), N17(2), N18(2), N19(2) | 0.8 | 0.005 |
| spa3 | C17(10) | 0 | 0 | 10 | N20(10) | 0 | 0 |
| spa4 | C17(9) | 0 | 0 | 9 | N20(9) | 0 | 0 |
| spa5 | C18(10) | 0 | 0 | 10 | N18(8), N21(2) | 0.356 | 0.002 |
| spa6 | C19(7) | 0 | 0 | 7 | N22(7) |  |  |
| spa7 | C20(10) | 0 | 0 | 10 | N15(2), N16(4), N18(2), N21(2) | 0.8 | 0.004 |
| spa8 | C20(7), C21(3) | 0.467 | 0.00053 | 10 | N15(8), N16(2) | 0.356 | 0.001 |
| spa9 | C22(8) | 0 | 0 | 8 | N16(8) | 0 | 0 |
| spa10 | C18(6), C23(2) | 0.429 | 0.00024 | 8 | N18(8) | 0 | 0 |
| spa11 | C15(8) | 0 | 0 | 8 | N15(8) | 0 | 0 |
| spa12 | C20(10) | 0 | 0 | 10 | N15(8), N18(2) | 0.356 | 0.004 |
| spa13 | C20(10) | 0 | 0 | 10 | N15(4), N16(2), N23(2), N24(2) | 0.8 | 0.002 |
| spa14 | C15(7), C24(3) | 0.467 | 0.00106 | 10 | N15(8), N16(2) | 0.356 | 0.001 |
| spa15 | C20(10) | 0 | 0 | 10 | N15(6), N16(2), N18(2) | 0.622 | 0.003 |
| spa16 | C20(5), C25(5) | 0.556 | 0.00032 | 10 | N15(6), N16(2), N25(2) | 0.622 | 0.001 |
| spa17 | C20(10) | 0 | 0 | 10 | N15(10) | 0 | 0 |
| spa18 | C20(4), C22(3), C26(4) | 0.727 | 0.00182 | 11 | N16(3), N26(2), N27(6) | 0.654 | 0.005 |
| spa19 | C18(10) | 0 | 0 | 10 | N18(10) | 0 | 0 |
| spa20 | C20(10) | 0 | 0 | 10 | N16(2), N18(6), N28(2) | 0.622 | 0.004 |
| spa21 | C20(7), C27(3) | 0.467 | 0.00027 | 10 | N18(2), N26(2), N29(4), N30(2) | 0.8 | 0.003 |
| spa22 | C20(10) | 0 | 0 | 10 | N18(4), N31(4), N32(2) | 0.711 | 0.002 |
| total |  | 0.78041 | 0.00177 | 211 |  | 0.8125 | 0.00541 |
| *C. thalictrifolium* |  |  |  |  |  |  |  |
| tha1 | C28(7), C29(3) | 0.467 | 0.00027 | 10 | N33(10) | 0 | 0 |
| tha2 | C30(7), C31(3) | 0.467 | 0.002 | 10 | N33(8), N34(2) | 0.356 | 0.001 |
| tha3 | C32(10) | 0 | 0 | 10 | N33(8), N35(2) | 0.356 | 0.001 |
| tha4 | C33(10) | 0 | 0 | 10 | N36(2), N37(6), N38(2） | 0.622 | 0.001 |
| tha5 | C34(8) | 0 | 0 | 8 | N33(8) | 0 | 0 |
| tha6 | C35(5), C36(5) | 0.556 | 0.00063 | 10 | N33(10) | 0 | 0 |
| tha7 | C33(7), C37(3) | 0.467 | 0.00027 | 10 | N36(4), N37(6) | 0.533 | 0.001 |
| tha8 | C38(8) | 0 | 0 | 8 | N37(8) | 0 | 0 |
| tha9 | C34(10) | 0 | 0 | 10 | N33(10) | 0 | 0 |
| total |  | 0.88153 | 0.00339 | 86 |  | 0.55157 | 0.00165 |
| *C. viridiflorum* |  |  |  |  |  |  |  |
| vir1 | C39(3), C40(3) | 0.6 | 0.001 | 6 | N39(4), N40(2) | 0.533 | 0.001 |
| vir2 | C41(6), C42(3) | 0.5 | 0.00085 | 9 | N41(9) | 0 | 0 |
| vir3 | C43(7), C44(3) | 0.467 | 0.00053 | 10 | N42(10) | 0 | 0 |
| vir4 | C45(6) | 0 | 0 | 6 | N43(6) | 0 | 0 |
| vir5 | C46(9) | 0 | 0 | 9 | N44(9) | 0 | 0 |
| total |  | 0.87308 | 0.0059 | 40 |  | 0.82179 | 0.00475 |
| *C. wolffianum* |  |  |  |  |  |  |  |
| wol1 | C47(5), C48(5) | 0.556 | 0.00031 | 10 | N45(6), N46(4) | 0.533 | 0.001 |
| wol2 | C49(8) | 0.25 | 0.00014 | 8 | N46(4), N47(4) | 0.571 | 0.001 |
| wol3 | C50(4), C51(3), C52(3) | 0.733 | 0.002 | 10 | N46(10) | 0 | 0 |
| total |  | 0.85979 | 0.00148 | 28 |  | 0.53968 | 0.00098 |
| all population |  | 0.94435 | 0.01543 | 525 |  | 0.937 | 0.029 |
